# Functional vitamin K insufficiency, vascular calcification and mortality in advanced chronic kidney disease: A cohort study

**DOI:** 10.1371/journal.pone.0247623

**Published:** 2021-02-24

**Authors:** Lu Dai, Longkai Li, Helen Erlandsson, Armand M. G. Jaminon, Abdul Rashid Qureshi, Jonaz Ripsweden, Torkel B. Brismar, Anna Witasp, Olof Heimbürger, Hanne Skou Jørgensen, Peter Barany, Bengt Lindholm, Pieter Evenepoel, Leon J. Schurgers, Peter Stenvinkel

**Affiliations:** 1 Division of Renal Medicine and Baxter Novum, Department of Clinical Science, Intervention and Technology, Karolinska Institutet, Stockholm, Sweden; 2 Department of Nephrology, First Affiliated Hospital of Dalian Medical University, Dalian, China; 3 Department of Biochemistry, Cardiovascular Research Institute Maastricht, Maastricht University, Maastricht, The Netherlands; 4 Division of Medical Imaging and Technology, Department of Clinical Science, Intervention and Technology, Karolinska Institutet, Stockholm, Sweden; 5 Department of Radiology, Karolinska University Hospital, Huddinge, Stockholm, Sweden; 6 Department of Microbiology Immunology and Transplantation, Nephrology and Renal Transplantation Research Group, KU Leuven-University of Leuven, Leuven, Belgium; 7 Department of Nephrology, University Hospitals Leuven, Leuven, Belgium; Kaohsiung Medical University Hospital, TAIWAN

## Abstract

Patients with chronic kidney disease (CKD) suffer from vitamin K deficiency and are at high risk of vascular calcification (VC) and premature death. We investigated the association of functional vitamin K deficiency with all-cause mortality and whether this association is modified by the presence of VC in CKD stage 5 (CKD G5). Plasma dephosphorylated-uncarboxylated matrix Gla-protein (dp-ucMGP), a circulating marker of functional vitamin K deficiency, and other laboratory and clinical data were determined in 493 CKD G5 patients. VC was assessed in subgroups by Agatston scoring of coronary artery calcium (CAC) and aortic valve calcium (AVC). Backward stepwise regression did not identify dp-ucMGP as an independent determinant of VC. During a median follow-up of 42 months, 93 patients died. Each one standard deviation increment in dp-ucMGP was associated with increased risk of all-cause mortality (sub-hazard ratio (sHR) 1.17; 95% confidence interval, 1.01–1.37) adjusted for age, sex, cardiovascular disease, diabetes, body mass index, inflammation, and dialysis treatment. The association remained significant when further adjusted for CAC and AVC in sub-analyses (sHR 1.22, 1.01–1.48 and 1.27, 1.01–1.60, respectively). In conclusion, functional vitamin K deficiency associates with increased mortality risk that is independent of the presence of VC in patients with CKD G5.

## Introduction

In patients with chronic kidney disease (CKD), the biological age of the arterial vasculature exceeds the chronological age [1, 2]. This premature vascular ageing phenotype, characterized by accelerated vascular calcification (VC), is highly prevalent in CKD and a strong predictor of cardiovascular morbidity and mortality [3, 4]. While VC is accentuated in advanced CKD, kidney transplantation may at best slow the progression [5, 6].

VC is a cell-mediated active process highly driven by vascular smooth muscle cells (VSMCs) with a complex involvement of mediators and effectors [7]. Matrix Gla protein (MGP) is a vitamin K dependent protein primarily synthesized and secreted by VSMCs [8]. MGP inhibits VC *in vivo* [9–11], possibly by directly binding the hydroxyapatite in the arterial walls [12] and by downregulating the function of bone morphogenetic proteins (BMP)-2 [13] and BMP-4 [14]. The activation of MGP requires two post-translational modifications, including serine phosphorylation and γ-glutamate carboxylation [15, 16]. Vitamin K serves as a cofactor for the carboxylation of MGP by converting glutamate residues into γ-carboxyglutamate [15, 17]. Vitamin K insufficiency, therefore, limits carboxylation of MGP in VSMCs, resulting in subsequent high secretion of dephosphorylated uncarboxylated MGP (dp-ucMGP). High circulating dp-ucMGP levels, indicative of functional vitamin K deficiency, have been associated with mortality in patients with CKD [18–20], diabetes [21], and cardiovascular disease (CVD) [22–24], and in the general population [25, 26]. Whether this association can be explained by its role as an inhibitor of VC is being debated. Observational association studies between functional vitamin K status and VC are ambiguous [27–31] and the recent K4Kidneys trial showed that vitamin K2 supplementation for 12 months failed to improve vascular stiffness or other measures of vascular health in patients with CKD [32]. More solid evidence is to be expected from ongoing randomized clinical trials (e.g. the VItaK-CAC, VitaVasK, the Aortic Valve DECalcification and BASIK2 trials) investigating the effect of vitamin K supplementation on VC progression.

Vitamin K deficiency is prevalent in CKD and progresses with the decline of renal function [18, 30, 33, 34]. However, data regarding the prevalence of vitamin K deficiency and its association with clinical outcome in patients with advanced CKD, defined as CKD G5, are limited. To further explore the clinically equivocal association between dp-ucMGP and VC, the present observational cohort study aimed to investigate the association between functional vitamin K deficiency and all-cause and cardiovascular mortality, and whether this association is modified by the presence of VC, evaluated by coronary artery calcium (CAC) and aortic valve calcium (AVC), in advanced CKD.

## Materials and methods

### Design and study population

Among 789 clinically stable adult (age >18 years) patients with stable CKD G5 receiving or referred for kidney replacement therapy who were enrolled in ongoing cohort studies at Karolinska University Hospital Huddinge between June 1996 and December 2016, 493 patients with baseline plasma dp-ucMGP measurement (obtained between October 2001 and October 2016) were included in this cross-sectional observational study with longitudinal follow-up. Thirty-four % (n = 170) were CKD G5D patients, of which 88 patients were scheduled for living donor kidney transplantation (LD-Rtx), while 66% (n = 323) were CKD G5 non-dialysis patients, of which 53 patients were scheduled for LD-Rtx. A subgroup of patients underwent computed tomography (CT) scan for the estimation of CAC (n = 237) and AVC (n = 223) in the frame of a study investigating the prevalence and natural history of VC in CKD. Patients were followed from inclusion until RTx or death, or until completing 60 months of follow-up. Causes of death were established by an epicrisis issued by the attending physician. The study was conducted in adherence to the Declaration of Helsinki and approved by the ethical committees of Karolinska University Hospital. A written informed consent was obtained from each patient.

Samples from age- and sex-matched samples of subjects (n = 467) with normal kidney function and no evidence of comorbidities or vitamin K antagonist use were collected and analyzed by the Department of Biochemistry, Maastricht University, the Netherlands, and were included as healthy controls for comparison with dp-ucMGP levels in CKD G5. A written informed consent was obtained from each healthy subject and samples were collected according to the guidelines laid down in the Declaration of Helsinki, and approved by the Medical Ethics Committee of the Maastricht University Medical Center (Maastricht, The Netherlands).

### Data collection

#### Clinical data

Relevant demographics, comorbidities, medications, and routine biochemistry were extracted from electronic files. CVD was defined based on clinical history or signs of ischemic cardiac disease, peripheral vascular disease, and/or cerebrovascular disease. Nutritional status was evaluated by 4-point scale subjective global assessment (SGA) to identify signs of malnutrition (defined as SGA score > 1) [35]. Handgrip strength (HGS) was measured using the Harpenden dynamometer (Yamar, Jackson, MI, USA) and repeated three times, with the largest value recorded and expressed in kg; for the statistical analyses, HGS was expressed as % of the mean HGS of healthy individuals of same sex. Cardiovascular mortality was defined as death due to coronary heart disease, sudden death, stroke or complicated peripheral vascular disease.

#### Biochemistry

Blood samples were taken at recruitment after an overnight fast. Samples were stored for <2 hours at 5°C until centrifugation. Upon arrival at the laboratory, the blood samples were centrifuged at 1900 g for 10 min, aliquoted, and either processed immediately (standard techniques) or stored at –80°C until analysis. Creatinine, albumin, hemoglobin, calcium, phosphate, total cholesterol (TC), triglyceride, high-density lipoprotein (HDL) and high-sensitivity C-reactive protein (hsCRP, high sensitivity nephelometric assay) were measured using standard methods at the Department of Laboratory Medicine, Karolinska University Hospital at Huddinge. Intact parathyroid hormone (PTH) was determined by an automated second-generation electro-chemiluminescence immunoassay on a Roche Cobas platform (Roche Diagnostics, GmbH, Germany).

#### Vitamin K status

Vitamin K status was indirectly evaluated by measuring plasma dp-ucMGP levels in a single run by the Laboratory of Coagulation Profile (Maastricht, the Netherlands) using the commercially available IVD CE-marked chemiluminescent InaKtif MGP assay on the IDS-iSYS system (IDS, Boldon, UK) [36]. In brief, plasma samples and internal calibrators were incubated with magnetic particles coated with murine monoclonal antibodies against dp-MGP, acridinium-labelled murine monoclonal antibodies against ucMGP and an assay buffer. The magnetic particles were captured using a magnet and washed to remove any unbound analyte. Trigger reagents were added, and the resulting light emitted by the acridinium label was directly proportional to the level of dp-ucMGP in the sample. The assay measuring range was between 300 and 12,000 pmol/L and was linear up to 11,651 pmol/L. The within-run and total variations of this assay were 0.8–6.2% and 3.0–8.2%, respectively.

#### CAC and AVC scoring

A subgroup of patients underwent non-contrast multi-detector cardiac CT (LightSpeed VCT or Revolution CT; GE Healthcare, Milwaukee, WI, USA) scanning with a standard ECG-gated protocol. CAC was assessed as a lesion with an area >1 mm^2^ and peak intensity >130 Hounsfield units (HU) based on the Agatston method, and the Agatston scores of each coronary artery were summed to determine the CAC score (total Agatston score). AVC was determined as the sum of total calcifications in the aortic valve area, including calcifications within the valve leaflets, as well as in the aortic wall immediately connected to the leaflets [37]. In patients with CAC scoring, we separately investigated CAC density and volume score. The area score (mm^2^) was calculated by dividing the CAC volume score (mm^3^) by slice thickness (2.5mm). CAC density score (score 1–4) was then calculated as the total CAC score (Agatston score) divided by the area score, representing the average calcified lesion density for all CT slices [38, 39].

### Statistical analysis

Data are expressed as median (interquartile range, IQR), mean (standard deviation, SD), or number and percentage, as appropriate. Statistical significance was set at the level of p <0.05. Comparisons between three groups were assessed with the non-parametric ANOVA Kruskal Wallis test for skewed continuous variables, one-way ANOVA for normally distributed variables and Fischer´s exact test for nominal variables. Spearman rank correlation analysis was used to determine correlations between dp-ucMGP, VC and other clinical and biochemical data. Multivariate linear and logistic regression analyses were performed to identify independent determinants of dp-ucMGP and presence of VC, respectively. Continuous variables were transformed to one standard deviation (1-SD) increments in linear and logistic regression analyses. Variables with statistical significance in the Spearman correlation analyses were first entered into a multivariate regression model and were then eliminated with a backward-selection estimation, where variables with p>0.05 were eligible for removal from the model. We used Fine and Gray models to evaluate the effect of high dp-ucMGP on mortality with kidney transplantation as a competing risk of all-cause death. The competing risk model was adjusted for age, sex, diabetes, CVD, body mass index (BMI), inflammation (hsCRP >10 mg/L) and dialysis treatment in all patients. To investigate the modification effect of VC on the association between dp-ucMGP and mortality, the model was performed with further adjustment for the presence of CAC and AVC in subgroups of patients where these measurements were available. Sub-analysis was also performed in dialysis and non-dialysis patients respectively as a sensitivity analysis. Statistical analyses were performed using Stata 16.1 (Stata Corporation, College Station, TX, USA). Figures were created using GraphPad Prism (version 8.0 GraphPad Software, www.graphpad.com).

## Results

### Study population characteristics

The main characteristics of 493 patients (63% of those enrolled in the original CKD cohorts) with baseline plasma dp-ucMGP measurement and therefore included in the present analysis are described in Table 1. Their median age was 55 years, 66% were men, median BMI was 24.6 (IQR 22.2, 27.9) kg/m^2^, 25% of the patients had diabetes, 30% had CVD, 29% showed signs of malnutrition (SGA score >1) and 34% were treated by dialysis. Additionally, 49% of the patients were treated with calcium channel blocker (CCB), 65% with beta-blocker, 65% with angiotensin-converting enzyme inhibitor / angiotensin receptor blocker (ACEi/ARB), 38% with statin, 54% with sevelamer and 4% with a vitamin K antagonist (VKA). Among patients who underwent cardiac CT, the median of CAC (n = 237) and AVC (n = 223) was 111 (IQR 0–1073) AU and 0 (IQR 0–39) AU, respectively.

**Table 1:** Baseline characteristics of CKD G5 patients according to tertiles of dp-ucMGP.

	Total	Low tertile	Middle tertile	High tertile	p-value
	N = 493	N = 163	N = 163	N = 167	
**Demography and clinical characteristics**					
Age, years	55 (44–65)	50 (40–61)	56 (42–64)	59 (49–69)	<0.0001
Male sex, n (%)	322 (66%)	105 (65%)	102 (63%)	115 (70%)	0.37
Diabetes, n (%)	120 (25%)	40 (25%)	32 (20%)	48 (29%)	0.13
CVD, n (%)	146 (30%)	38 (23%)	44 (27%)	64 (39%)	0.007
Systolic BP, mmHg	144 (130–159)	144 (130–156)	145 (132–157)	142 (129–162)	0.87
Diastolic BP, mmHg	85 (76–92)	86 (78–92)	85 (75–94)	82 (75–92)	0.15
CKD G5D, n (%)	170 (34%)	35 (21%)	61 (37%)	74 (44%)	<0.0001
**Nutritional status**					
Malnutrition (SGA>1)	140 (29%)	42 (26%)	41 (26%)	57 (35%)	0.12
BMI, kg/m^2^	24.6 (22.2–27.9)	23.8 (21.5–27.0)	24.2 (22.0–27.9)	25.4 (23.4–28.4)	<0.0001
HGS%	84 (67–100)	89 (70–104)	85 (67–102)	77 (63–95)	0.007
**Biochemical markers**					
Hemoglobin, g/L	110 (101–119)	109 (99–117)	112 (101–120)	110 (102–120)	0.30
Albumin, g/L	34 (31–37)	35 (31–37)	34 (31–37)	33 (30–36)	0.008
Cholesterol, mmol/L	4.5 (3.8–5.3)	4.4 (3.8–5.3)	4.6 (3.8–5.2)	4.5 (3.7–5.3)	0.77
HDL, mmol/L	1.3 (1.0–1.6)	1.4 (1.1–1.7)	1.2 (1.0–1.5)	1.3 (1.0–1.6)	0.01
Triglycerides, mmol/L	1.5 (1.1–2.2)	1.4 (1.0–1.9)	1.5 (1.1–2.4)	1.6 (1.2–2.2)	0.02
Calcium, mmol/L	2.3 (2.2–2.5)	2.4 (2.2–2.5)	2.3 (2.2–2.5)	2.3 (2.2–2.5)	0.008
Phosphate, mmol/L	1.8 (1.5–2.2)	1.8 (1.5–2.1)	1.8 (1.4–2.4)	1.8 (1.5–2.1)	0.59
iPTH, ng/L	250 (138–392)	218 (120–358)	259 (141–394)	271 (151–405)	0.07
Creatinine, μmol/L	732 (594–888)	705 (574–907)	743 (590–891)	738 (610–880)	0.71
hsCRP, mg/L	2.4 (0.8–8.3)	1.6 (0.6–6.4)	1.9 (0.8–7.5)	3.9 (1.2–10.6)	<0.0001
dp-uc MGP, pmol/L	1445 (932–2048)	763 (468–914)	1424 (1246–1573)	2473 (2011–3283)	<0.0001
**CAC and AVC** *					
CAC score, AU	111 (0–1073)	10 (0–392)	61 (0–1042)	485 (25–1767)	<0.0001
CAC density score	3.17 (3.03–3.29)	3.15 (2.89–3.29)	3.15 (2.98–3.27)	3.20 (3.07–3.30)	0.33
CAC volume, mm^3^	67 (0–803)	6 (0–418)	47 (0–803)	284 (20–1222)	0.0006
AVC score, AU	0 (0–39)	0 (0–2)	0 (0–27)	5 (0–59)	0.01
**Medications**					
CCB, n (%)	216 (49%)	76 (50%)	75 (51%)	65 (46%)	0.66
Beta-blocker, n (%)	285 (65%)	90 (59%)	92 (63%)	103 (73%)	0.03
ACEi/ARB, n (%)	291 (65%)	100 (63%)	98 (66%)	93 (66%)	0.75
Statin, n (%)	169 (38%)	46 (29%)	59 (40%)	64 (45%)	0.01
Sevelamer, n (%)	265 (54%)	85 (52%)	98 (60%)	82 (49%)	0.12
VKA, n (%)	21 (4%)	1 (0.6%)	2 (1%)	18 (11%)	<0.0001

Abbreviations: CKD G5, chronic kidney disease stage 5; dp-ucMGP, dephosphorylated-uncarboxylated matrix-Gla protein; CVD, cardiovascular disease; BP, blood pressure; CKD G5D, chronic kidney disease stage 5 treated by dialysis; SGA, subjective global assessment; BMI, body mass index; HGS%, hand grip strength, converted to % of sex-matched healthy controls; HDL, high-density lipoprotein; iPTH, intact parathyroid hormone; hsCRP, high sensitivity C-reactive protein; CAC, coronary artery calcium; AVC, aortic valve calcium; AU, Agatston units; CCB, calcium channel blockers; ACEi/ARB, angiotensin-converting enzyme inhibitor/ angiotensin II receptor blocker; VKA, vitamin K antagonist.

* CAC available among 237 patients, AVC available among 223 patients.

### Functional vitamin K status

#### Dp-ucMGP in healthy subjects and CKD G5

**Fig 1:**
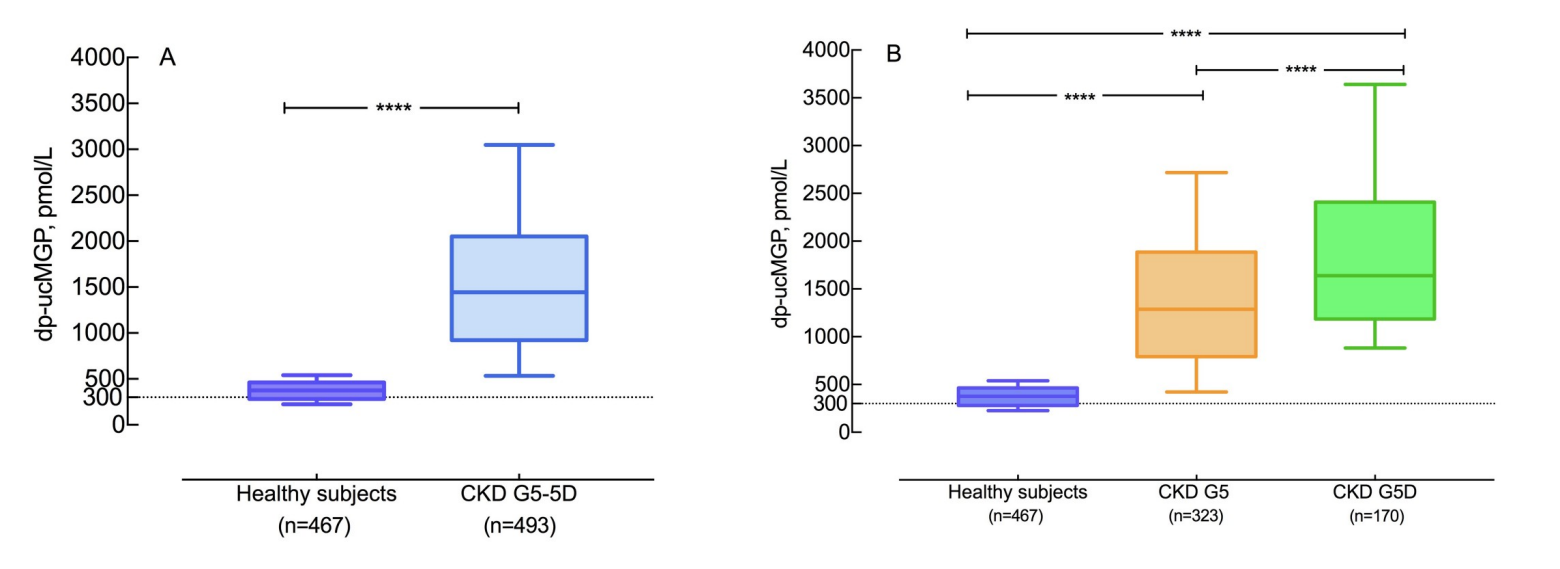
Plasma dp-ucMGP in healthy subjects and CKD G5 patients. (A) Distribution of dp-ucMGP in healthy subjects and CKD G5-5D patients illustrated by box plots where whiskers denote 10%-90% percentile range; (B) Distribution of dp-ucMGP in healthy subjects and subgroups of CKD G5 patients with and without kidney replacement therapy respectively. Dp-ucMGP <300 pmol/L is considered as normal reference; ****, p<0.0001; Abbreviations: CKD G5-5D, chronic kidney disease stage 5 treated or not treated by dialysis; CKD G5, chronic kidney disease stage 5 not treated by dialysis; CKD G5D, chronic kidney disease stage 5 treated by dialysis.

Compared to age and sex-matched healthy controls, dp-ucMGP was increased in CKD G5 (median (IQR) 1445 (932, 2048) pmol/L vs. 377 (281, 464) pmol/L) (Fig 1A and 1B). As expected, dp-ucMGP was significantly higher among the 21 patients prescribed VKA vs. not treated with VKA (S1 Fig).

Patients were further classified according to tertiles of dp-ucMGP (Table 1). Patients in the highest tertile were older, had more often CVD, were more often on dialysis treatment, had higher BMI and lower %HGS—and they were characterized by dyslipidemia with lower HDL and higher triglyceride as well as a high inflammatory burden with lower albumin and higher hsCRP. Additionally, these patients were more commonly prescribed beta-blockers, statins and VKAs. Patients with higher dp-ucMGP presented with higher total CAC, higher CAC volume score and total AVC.

#### Determinants of dp-ucMGP

Univariate correlations between dp-ucMGP and other variables are described in S1 Table (p<0.05 presented). To identify independent factors associated with dp-ucMGP, we performed multivariate linear regression analyses. Table 2 shows that older age, presence of malnutrition, high BMI, dialysis treatment, and VKA use were independent determinants of high dp-ucMGP in the overall cohort (adjusted r^2^ = 0.33). We also performed sensitivity analysis on determinants of dp-ucMGP in the subgroups with VC assessment. Using backward stepwise regression, older age, high BMI, VKA and CCB were selected as determinants of dp-ucMGP in CAC and AVC subgroups (adjusted r^2^ = 0.34, S3 Table). Neither the presence of CAC nor AVC were independently associated with high dp-ucMGP.

**Table 2:** Determinants of dp-ucMGP in 493 CKDG5 patients*.

	per 1-SD increase of dp-ucMGP,	
	Model pseudo r^2^ = 0.33, p<0.0001	
	Coefficients	p-value
Age, per 1-SD increase	0.16	<0.0001
Malnutrition (SGA>1), yes/no	0.22	0.02
BMI, per 1-SD increase	0.15	0.001
Dialysis treatment, yes/no	0.37	<0.0001
Vitamin K antagonist use, yes/no	2.30	<0.0001

Abbreviations: dp-ucMGP, dephosphorylated-uncarboxylated matrix-Gla protein; SD, standard deviation; SGA, subjective global assessment; BMI, body mass index.

* Multivariate linear regression with stepwise backward selection of variables.

When CAC score was separated into CAC volume and CAC density score, we found that CAC volume, but not CAC density, was correlated with dp-ucMGP in univariate analysis (S1 Table). However, the subsequent sensitivity analysis with CAC volume score 0 as reference, showed that neither lower nor higher median CAC volume score were determinants of dp-ucMGP (S3 Table).

### Functional vitamin K status and VC

**Fig 2:**
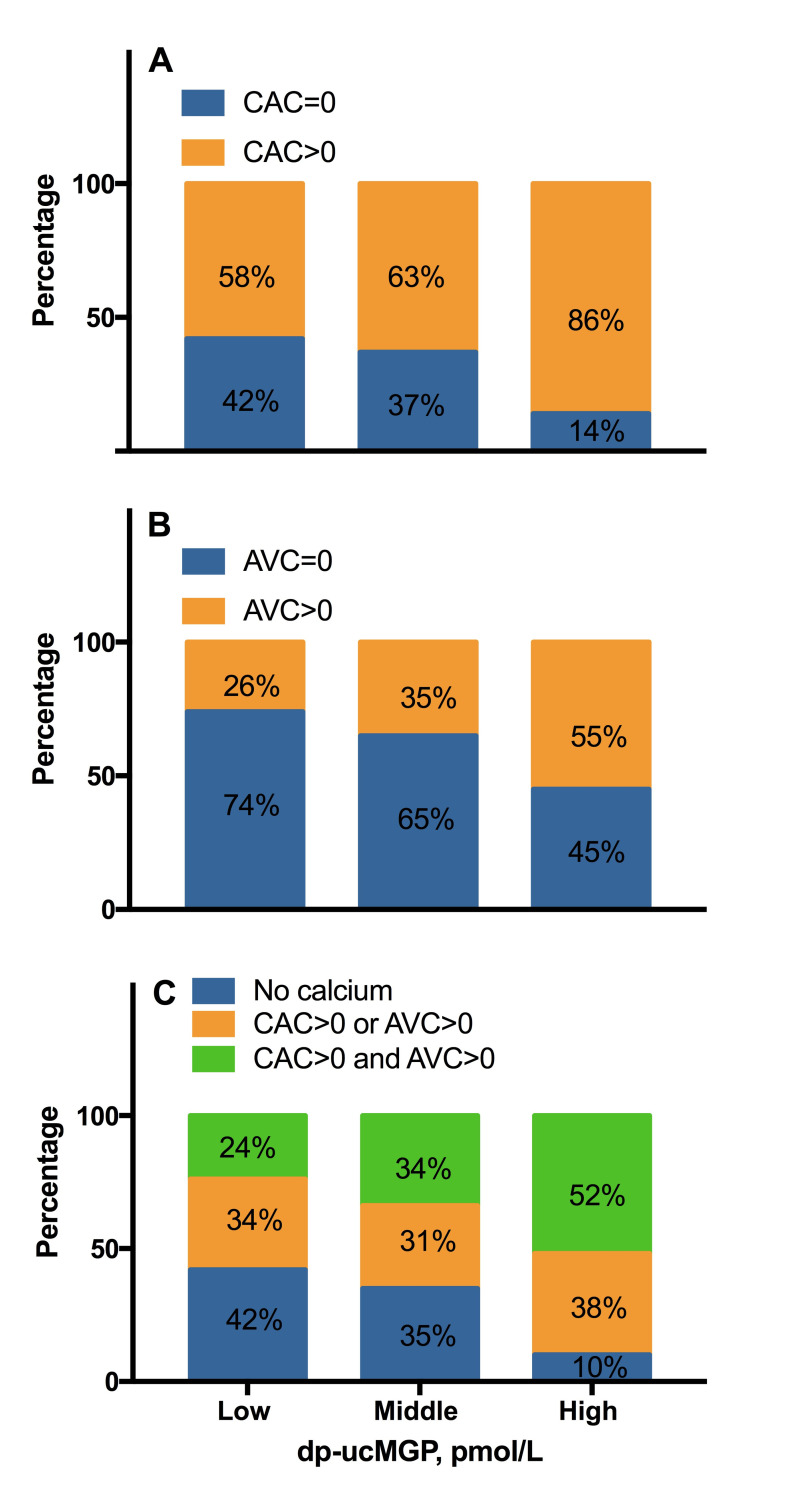
Prevalence of vascular calcification according to tertiles of dp-ucMGP. **(A)** Prevalence of CAC across tertiles of dp-ucMGP (n = 237). **(B)** Prevalence of AVC across tertiles of dp-ucMGP (n = 223). **(C)** Prevalence of combined prevalence of CAC and AVC across tertiles of dp-ucMGP (n = 237). Abbreviations: dp-ucMGP, dephosphorylated-uncarboxylated matrix-Gla protein; CAC, coronary artery calcium; AVC, aortic valve calcium.

Patients with highest dp-ucMGP presented with highest total CAC score, CAC volume score and total AVC score (Table 1), and the proportion of patients with presence of CAC and AVC increased across tertiles of dp-ucMGP, from 58% to 86% (p = 0.0001) and from 26% to 55% (p = 0.001), respectively (Fig 2A and 2B). The same trend was observed for the combined presence of CAC and AVC (p<0.0001). The presence of calcification at one site (CAC >0 or AVC>0) or at both sites (CAC >0 and AVC>0) increased with elevated dp-ucMGP levels, reaching 38% and 52% in the highest dp-ucMGP tertile, respectively (Fig 2C).

As expected, dp-ucMGP was significantly correlated with CAC and AVC in Spearman’s rank correlation analyses (S2 Table). However, the backward stepwise regression model showed that dp-ucMGP was not an independent determinant of CAC or AVC; instead determinants of CAC included older age, and diabetes (pseudo r^2^ = 0.45) and determinants of AVC were older age, lower albumin and beta-blocker use (pseudo r^2^ = 0.29) (Table 3).

**Table 3:** Determinants of the presence of vascular calcification*.

	Presence of CAC		Presence of AVC	
	(*n* = 237, pseudo r^2^ = 0.45)		(*n =* 223, pseudo r^2^ = 0.29)	
	OR	95% CI	OR	95% CI
Age, per 1-SD increase	8.10	4.43–14.81	3.83	2.39–6.14
Diabetes, yes/no	10.49	1.32–83.18	-	-
Beta-blocker, yes/no	-	-	2.23	1.03–4.78
Albumin, per 1-SD increase	-	-	0.59	0.41–0.85

Abbreviations: CAC, coronary artery calcium; AVC, aortic valve calcium; OR, odds ratio; CI, confidence interval.

* Multivariate logistic regression with stepwise backward selection of variables.

### Functional vitamin K status and mortality

#### Functional vitamin K status and all-cause mortality

**Fig 3:**
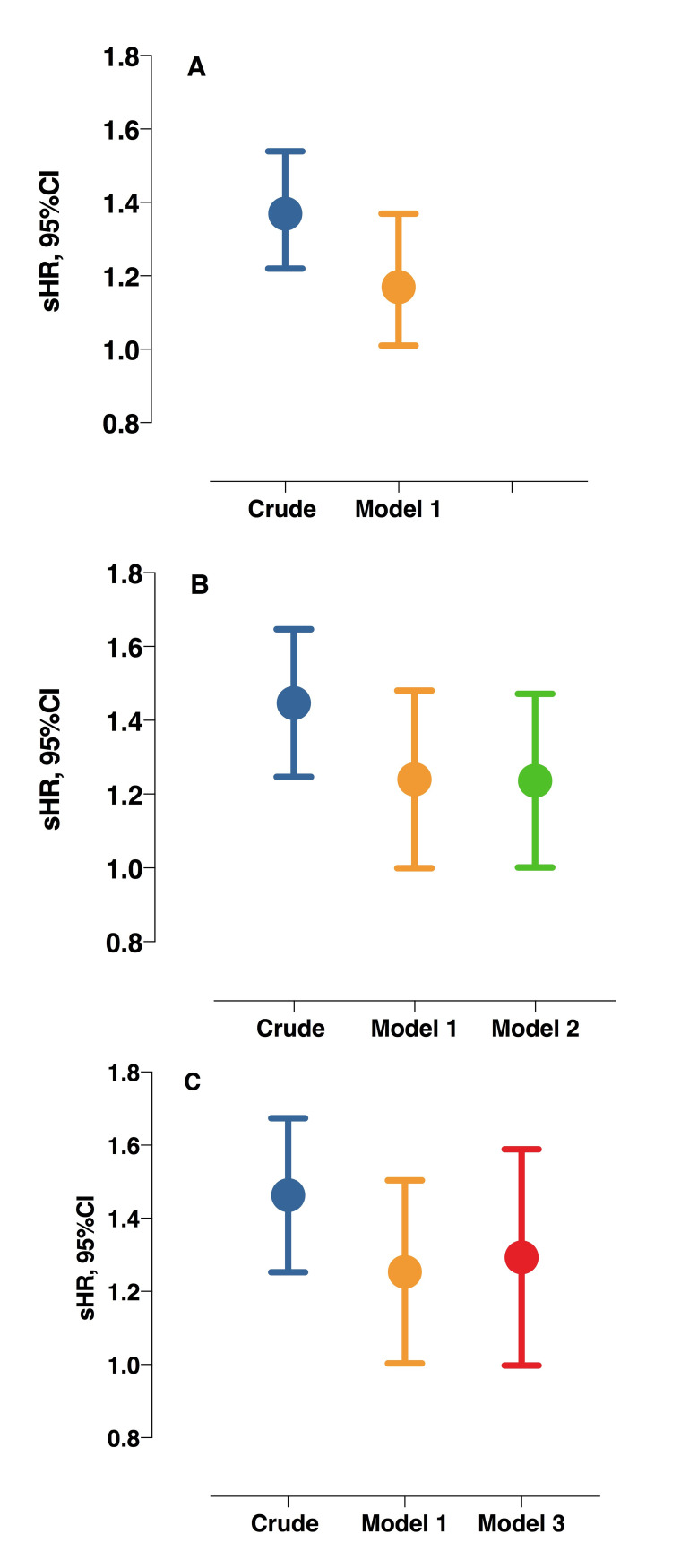
Associations of dp-ucMGP with all-cause mortality in all patients (A, n = 493) and sub-group of patients with assessment of CAC (B, n = 237) and AVC (C, n = 223). Model 1, adjusted for age, sex, cardiovascular disease, diabetes, body mass index, inflammation, and dialysis treatment; Model 2: Model 1 plus presence of CAC; Model 3, model 1 plus presence of AVC. Abbreviations: sHR, sub-hazard ratio; CI, confidence interval.

To investigate the effect of functional vitamin K deficiency on clinical outcome, we first investigated the association between dp-ucMGP and all-cause mortality. During a median follow-up of 42 months, 93 patients (19%) died and 128 patients (26%) underwent RTx. In the crude model, each 1-SD increment in plasma dp-ucMGP associated with increased risk of all-cause mortality (sub-hazard ratio (sHR) 1.37; 95% confidence interval (95% CI), 1.22–1.54) and this association withstood multivariate adjustment for age, sex, CVD, diabetes, BMI, inflammation, and dialysis treatment (sHR 1.17, 95%CI 1.01–1.37) (Fig 3A).

To investigate whether the association between dp-ucMGP and mortality could withstand the adjustment for presence of VC, we performed further survival analyses in subgroups of patients with available data on CAC and AVC, using the same construction of models as previously. Among patients with CAC scoring (n = 237), both the crude and adjusted models (adjusted for age, sex, CVD, diabetes, BMI, inflammation and dialysis treatment), showed sustained significant associations between dp-ucMGP and all-cause mortality (sHR 1.44, 95%CI 1.25–1.65 and sHR 1.22, 95%CI 1.01–1.49, respectively) (Fig 3B). Moreover, with further adjustment for presence of CAC, each 1-SD increment in dp-ucMGP remained independently associated with increased risk of all-cause mortality (sHR 1.22, 95%CI 1.01–1.48, Fig 3B).

In line with the sustained association between dp-ucMGP and mortality when adjusting also for CAC, a similar result was observed among patients with AVC (n = 223). Higher dp-ucMGP was associated with increased all-cause mortality in crude and adjusted models (sHR 1.45, 95%CI 1.26–1.68 and sHR 1.24, 95%CI 1.01–1.51, respectively) and this association remained significant also with further adjustment for the presence of AVC (sHR 1.27, 95%CI 1.01–1.60) (Fig 3C).

We also tested the association between dp-ucMGP and all-cause mortality in subgroups of patients with and without dialysis as sensitivity analyses. In the sub-group of dialysis patients, dp-ucMGP remained significantly associated with all-cause mortality in the unadjusted models, in models adjusted for age, sex, CVD, diabetes, BMI and inflammation, and in models further adjusted for CAC and AVC (S2 Fig). In the subgroup of non-dialysis patients, dp-ucMGP was significantly associated with increased mortality risk only in unadjusted model in all non-dialysis patients (S3A Fig), but not in the subgroup of non-dialysis patients with CAC or AVC measurement, nor in any of the adjusted models (S3B and S3C Fig).

#### Functional vitamin K status and cardiovascular mortality

We then investigated the association between dp-ucMGP and cardiovascular mortality among all patients and—as sensitivity analyses—in the subgroups with available assessment of VC. Out of 93 deaths, 43 were identified as CVD-related deaths. In the crude model, among all 493 patients, each 1-SD increment in plasma dp-ucMGP associated with increased risk of cardiovascular mortality (sHR 1.36; 95% CI, 1.16–1.59), but the association disappeared after adjustments for age, sex, CVD, diabetes, BMI, inflammation, and dialysis treatment (sHR 1.19, 95%CI 0.90–1.55) (S4A Fig).

In subgroups of patients with VC assessment, dp-ucMGP was significantly associated with cardiovascular mortality in patients with CAC and AVC but only in crude models (sHR 1.37, 95%CI 1.14–1.63 and sHR 1.39, 95%CI 1.15–1.67, respectively), whereas no significant associations were found after adjustments for age, sex, CVD, diabetes, BMI, inflammation and dialysis treatment, or with further adjustments for CAC or AVC (S4B and S4C Fig).

## Discussion

We report that high plasma dp-ucMGP concentrations (indicating functional vitamin K insufficiency) is highly prevalent and associates with all-cause mortality in patients with CKD G5. However, dp-ucMGP was not an independent determinant of VC assessed by CAC and AVC, and the association between dp-ucMGP and all-cause mortality was not modified by the presence of VC.

Vitamin K deficiency in CKD can be explained by a combination of factors such as dietary restrictions [40], impaired vitamin K recycling [41] and disturbed microbial metabolism [42]. In our study, the CKD G5 patients had about four times higher dp-ucMGP levels than age- and sex-matched healthy subjects, confirming that severe vitamin K deficiency is a typical feature of the uremic phenotype. Our observation that dp-ucMGP is strongly linked to presence of calcification at two different sites, represented by CAC and AVC, and the concomitant presence of calcification at both sites, extends previous reports of associations between dp-ucMGP and VC documented in various study populations [18, 28, 30, 33, 34, 43, 44]. Nevertheless, our stepwise regression analyses revealed that dp-ucMGP was not an independent determinant of VC represented by CAC and AVC and *vice versa;* neither CAC nor AVC were identified as determinants of dp-ucMGP in backward stepwise regression modeling. This seems contradictive to the observed rough correspondence between the distributions of presence of VC and plasma dp-ucMGP (Fig 2). It is worthwhile to mention, that although the role of MGP in VC has been well established in animal models [9–11], data are inconsistent in the clinical context. While some observational studies showed a positive association between circulating dp-ucMGP and VC [18, 28, 30, 43], others have failed to do so [27, 31, 44–46]. One possible explanation of these conflicting data lies in the heterogeneity of study populations, calcification measurements, and statistic methodology. In a randomized trial of 452 older adults assigned to receive multivitamins with or without phylloquinone, the vitamin K supplement group had less CAC progression, yet higher serum MGP, and the effect of phylloquinone on CAC progression was independent of the change in serum MGP [44]. To improve the problematic interpretation of total serum MGP levels, which do not differentiate between γ-carboxylation variants of MGP, the authors of that study re-investigated dp-ucMGP from archived blood samples. Their *post hoc* analysis showed that dp-ucMGP was not associated with CAC at baseline and the decrease of dp-ucMGP after three years of vitamin K supplementation was not associated with the change in CAC. The observation that although dp-ucMGP is a good marker of vitamin K status it does not predict CAC [46] concurs with the results from our stepwise regression showing that dp-ucMGP was not an independent determinant of neither CAC nor AVC. One reason could be that circulating dp-ucMGP at one single observational point does not reflect MGP expression in the calcified areas, as observed both in mice [47] and humans [48], and thus, fails to associate with the actual calcification present in coronary arteries and aortic valves. Also, the probable contrast between high dp-ucMGP turn-over and slow development of VC is difficult to reconcile with an affirmative association between dp-ucMGP and VC. Prospective studies evaluating the dynamic changes of dp-ucMGP and VC progression with multiple time windows are warranted to fill this gap in knowledge. Another unrevealed matter is the potential role of dp-ucMGP in favoring plaque mass or stability in the development of VC. Zwakenberg *et al* [48] showed that circulating dp-ucMGP was not associated with calcification levels in carotid plaque samples, but inversely associated with plaque hemorrhage. In addition, our data did not support an independent association between dp-ucMGP and CAC density score or CAC volume. Taken together, it is plausible that dp-ucMGP is more inclined to reflect active calcification (i.e., microcalcification) in vulnerable atherosclerotic plaques than the total mass of macrocalcification (i.e., volume and density) detected by conventional CT. Further studies investigating the role of dp-ucMGP in determining micro- and macrocalcification are warranted to elucidate its role in the evolution of plaque remodeling and calcification.

In agreement with previous studies supporting functional vitamin K deficiency as a risk factor of mortality and cardiovascular events in various populations [18–25, 49], we confirmed that higher dp-ucMGP is independently associated with increased risk of all-cause mortality in CKD G5 patients, after adjustments for age, sex, BMI, CVD, diabetes, inflammation, and dialysis treatment. Furthermore, this association withstood additional adjustment for the presence of VC, lending support to the hypothesis that functional vitamin K insufficiency may influence clinical outcome in CKD through processes independent of VC. Indeed, vitamin K-dependent proteins may exert anti-inflammatory effects, and recent studies revealed the multifunctional capacity of vitamin K including antioxidant and anti-inflammatory functions, independent of its role as cofactor for γ-glutamyl carboxylase [50, 51]. Hence, vitamin K might be beneficial for health and prevent aging via various pathways. A large body of observational and interventional studies implicate involvement of vitamin K deficiency in non-cardiovascular age-related disease phenotypes including frailty [52], physical decline [53], depression [54], osteoporosis [55] and fractures [56, 57]. Moreover, since vitamin K is involved in both senescence [58] and cancer progression [59], vitamin K deficiency may merely be a feature of “diseasome of aging” [60]. Since diet is the main source of vitamin K and its body stores are depleted within a few days, vitamin K deficiency may reflect poor health in general. A recent meta-analysis incorporating data from three large cohorts showed that low circulating phylloquinone concentrations (a direct measurement of vitamin K) were associated with an increased risk of all-cause mortality, yet not with CVD [61]. Our study corroborates these data, as we found that circulating dp-ucMGP was not a determinant of VC, nor was the association between dp-ucMGP and all-cause mortality modified by the presence of VC. The underlying mechanism(s) involved in the interplay between vitamin K deficiency and poor prognosis in CKD remains to be clarified.

Some strengths and limitations of this study should be noted. A major strength is the availability of measurements of dp-ucMGP, CAC, and AVC, together with clinical profiles in a large and well-phenotyped CKD G5 cohort with long-term follow-up. Our study is however limited by its observational design and as such it cannot establish causal relationships; thus we are not able to explain the ambiguous link between dp-ucMGP and VC. Secondly, vitamin K status was evaluated indirectly by measurement of plasma dp-ucMGP levels, and not by a direct measurement of circulating phylloquinone concentrations or other vitamin K markers. Though dp-ucMGP may not represent a true reflection of vitamin K status, it is likely to be associated with functional vitamin K deficiency involved in a multifaceted diseasome pathogenesis, whereas the direct measurement of phylloquinone serves as a more proximate reflection of diet and lifestyle. Studies differentiating the role of absolute and functional vitamin K deficiency in clinical outcomes are warranted. Thirdly, dp-ucMGP and VC were measured at a single time point at baseline. Repeated measures over time would be more reflective of the long-term interplay between dp-ucMGP and VC but were not available. Fourthly, we acknowledge the possibility of residual confounding as well as the undermined power issue in sample-decreased sub-analyses. Nevertheless, survival analyses with careful adjustments in all patients, and repeated as sensitivity analyses among VC sub-groups using the same model, confirm and reinforce that there is an independent association between high plasma dp-ucMGP concentrations and increased mortality in CKD G5 patients.

## Conclusions

Functional vitamin K deficiency is prevalent and strongly associated with an increased all-cause mortality risk in CKD G5. Plasma dp-ucMGP appears not to be an independent risk factor of calcification as represented by CAC and AVC. As such, our data highlight the importance of elucidating the exact role of vitamin K deficiency in the etiology of cardiovascular health, and more importantly, to reveal the mechanisms for its less explored function as a risk factor for non-cardiovascular causes of poor outcomes in CKD.

## Supporting information

S1 Fig Vitamin K antagonist use and plasma dp-ucMGP. (DOCX)

S2 Fig Associations of dp-ucMGP with all-cause mortality in dialysis patients (A, n = 170) and sub-group of patients with CAC (B, n = 118) and AVC (C, n = 114) assessment. Model 1, adjusted for age, sex, cardiovascular disease, diabetes, body mass index and inflammation; Model2, model 1 plus presence of CAC; Model 3, model 1 plus presence of AVC. Abbreviations: sHR, sub-hazard ratio; CI, confidence interval.
(DOCX)

S3 Fig Associations of dp-ucMGP with all-cause mortality in non-dialysis CKD G5 patients (A, n = 323) and sub-group of patients with CAC (B, n = 119) and AVC (C, n = 109) assessment. Model 1, adjusted for age, sex, cardiovascular disease, diabetes, body mass index and inflammation; Model2, model 1 plus presence of CAC; Model 3, model 1 plus presence of AVC. Abbreviations: sHR, sub-hazard ratio; CI, confidence interval.
(DOCX)

S4 Fig Associations of dp-ucMGP with cardiovascular mortality in all CKD G5 patients (A, n = 493) and sub-group of patients with CAC (B, n = 237) and AVC (C, n = 223) assessment. Model 1, adjusted for age, sex, cardiovascular disease, diabetes, body mass index, inflammation and dialysis treatment; Model2, model 1 plus presence of CAC; Model 3, model 1 plus presence of AVC. Abbreviations: sHR, sub-hazard ratio; CI, confidence interval.
(DOCX)

S1 Table Spearman rank correlations between dp-ucMGP and other variables (p<0.05 presented). (DOCX)

S2 Table Spearman rank correlations between CAC, AVC and other variables (p<0.05 presented). (DOCX)

S3 Table Multivariate linear regression of factors associated with per 1-SD increase of dp-ucMGP in subgroup patients (backward stepwise selection). (DOCX)

S1 Data Database file of demographic and biochemical features. (DAT)
