# Supplementary material for: Functional vitamin K insufficiency, vascular calcification and mortality in advanced chronic kidney disease: A cohort study
Source: PLoS One. 2021 Feb 24;16(2):e0247623. doi: 10.1371/journal.pone.0247623 (PMC7904143; doi:10.1371/journal.pone.0247623)
Supplement: S1 Fig — (DOCX) [file pone.0247623.s001.docx]

**S1 Fig. Vitamin K antagonist use and plasma dp-ucMGP**

Abbreviations: dp-ucMGP, dephosphorylated-uncarboxylated matrix-Gla protein, VitK, vitamin K

****, p<0.0001.
