# Supplementary material for: Functional vitamin K insufficiency, vascular calcification and mortality in advanced chronic kidney disease: A cohort study
Source: PLoS One. 2021 Feb 24;16(2):e0247623. doi: 10.1371/journal.pone.0247623 (PMC7904143; doi:10.1371/journal.pone.0247623)
Supplement: S4 Fig — Model 1, adjusted for age, sex, cardiovascular disease, diabetes, body mass index, inflammation and dialysis treatment; Model2, model 1 plus presence of CAC; Model 3, model 1 plus presence of AVC. Abbreviations: sHR, sub-hazard ratio; CI, confidence interval. (DOCX) [file pone.0247623.s004.docx]

**S4 Fig. Associations of dp-ucMGP with cardiovascular mortality in all CKD G5 patients (A, n=493) and sub-group of patients with CAC (B, n=237) and AVC (C, n=223) assessment**. Model 1, adjusted for age, sex, cardiovascular disease, diabetes, body mass index, inflammation and dialysis treatment; Model 2, model 1 plus presence of CAC; Model 3, model 1 plus presence of AVC. Abbreviations: sHR, sub-hazard ratio; CI, confidence interval
