# Supplementary material for: Functional vitamin K insufficiency, vascular calcification and mortality in advanced chronic kidney disease: A cohort study
Source: PLoS One. 2021 Feb 24;16(2):e0247623. doi: 10.1371/journal.pone.0247623 (PMC7904143; doi:10.1371/journal.pone.0247623)
Supplement: S1 Table — (DOCX) [file pone.0247623.s005.docx]

**S1 Table. Spearman rank correlations between dp-ucMGP and other variables (p<0.05 presented)**

|  | **All patients**  **(n=493)** | | **Subgroup patients with CAC^#^ (n=237)** | | **Subgroup patients with AVC ^#^ (n=223)** | |
| --- | --- | --- | --- | --- | --- | --- |
|  | **Rho** | **p value** | **Rho** | **p value** | **Rho** | **p value** |
| Age, years | 0.2660 | <0.0001 | 0.3901 | <0.0001 | 0.3848 | <0.0001 |
| Male sex | - | - | - | - | - | - |
| Diabetes | - | - | - | - | - | - |
| CVD | 0.1495 | 0.0009 | 0.1886 | 0.004 | 0.1635 | 0.01 |
| Systolic BP, mmHg | - | - | - | - | - | - |
| Diastolic BP, mmHg | -0.0939 | 0.04 | - | - | - | - |
| Dialysis treatment | 0.2232 | <0.0001 | - | - | - | - |
| SGA>1 | 0.0945 | 0.04 | - | - | - | - |
| BMI, kg/m2 | 0.1998 | <0.0001 | 0.2553 | <0.0001 | 0.2548 | 0.0001 |
| HGS % | -0.1401 | 0.002 | -0.2239 | 0.0006 | -0.2160 | 0.001 |
| Haemoglobin, g/L | - | - | - | - | - | - |
| Albumin, g/L | -0.1249 | 0.005 | - | - | - | - |
| Cholesterol, mmol/L | - | - | - | - | - | - |
| HDL, mmol/L | -0.1036 | 0.02 | -0.1448 | 0.03 | - | - |
| Triglycerides, mmol/L | 0.1420 | 0.002 | 0.1995 | 0.0020 | 0.1895 | 0.005 |
| Calcium, mmol/L | -0.0976 | 0.03 | - | - | - | - |
| Phosphate, mmol/L | - | - | - | - | - | - |
| iPTH, ng/L | - | - | - | - | - | - |
| Creatinine, μmol/L | - | - | - | - | - | - |
| hsCRP, mg/L | 0.1591 | 0.0004 | 0.3676 | <0.0001 | 0.3540 | <0.0001 |
| CCB | - | - | -0.1629 | 0.02 | -0.1830 | 0.008 |
| Betablock | 0.1184 | 0.01 | 0.1534 | 0.02 | 0.1376 | 0.05 |
| ACEi/ARB | - | - | - | - | - | - |
| Statin | 0.1401 | 0.003 | - | - | - | - |
| sevelamer | - | - | - | - | - | - |
| Warfarin | 0.2521 | <0.0001 | 0.2498 | 0.0001 | 0.2356 | 0.0004 |
| CAC >0 |  |  | 0.3100 | <0.0001 | 0.3040 | <0.0001 |
| CAC density score |  |  | - | - | - | - |
| CAC volume^*^, mm^3^ |  |  | 0.2832 | <0.0001 | 0.2832 | <0.0001 |
| AVC >0 |  |  | 0.2396 | 0.0003 | 0.2396 | 0.0003 |

Abbreviations: dp-ucMGP, dephosphorylated-uncarboxylated matrix-Gla protein; CAC, coronary artery calcium; AVC, aortic valve calcium; CVD, cardiovascular disease; BP, blood pressure; SGA, subjective global assessment; BMI, body mass index; HGS%, hand grip strength, converted to % of sex-matched healthy controls; HDL, high-density lipoprotein; iPTH, intact parathyroid hormone; hsCRP, high sensitivity C-reactive protein; AU, Agatston units; CCB, calcium channel blockers; ACEi/ARB, angiotensin-converting enzyme inhibitor/ angiotensin II receptor blocker

^#^ CAC and AVC were calculated by Agatston scoring

^*^ CAC volume was treated as an ordinal variable (CAC volume 0 as reference, lower than median CAC volume and higher than median CAC volume)
