# Supplementary material for: Functional vitamin K insufficiency, vascular calcification and mortality in advanced chronic kidney disease: A cohort study
Source: PLoS One. 2021 Feb 24;16(2):e0247623. doi: 10.1371/journal.pone.0247623 (PMC7904143; doi:10.1371/journal.pone.0247623)
Supplement: S2 Table — (DOCX) [file pone.0247623.s006.docx]

**S2 Table. Spearman rank correlations between CAC, AVC and other variables (p<0.05 presented)**

|  | **CAC>0 (n=237)^*^** | | **AVC>0 (n=223) ^*^** | |
| --- | --- | --- | --- | --- |
|  | **Rho** | **p value** | **Rho** | **p value** |
| Age, years | 0,6130 | <0,0001 | 0,5217 | <0,0001 |
| Male sex | - | - | - | - |
| Diabetes | 0,2826 | <0,0001 | 0,2741 | <0,0001 |
| CVD | 0,2226 | 0,0006 | 0,1973 | 0,0031 |
| Systolic BP, mmHg | - | - | - | - |
| Diastolic BP, mmHg | - | - | - | - |
| Dialysis treatment | - | - | - | - |
| SGA>1 | - | - | - | - |
| BMI, kg/m2 | 0,2301 | 0,0004 | 0,1940 | 0,0037 |
| HGS % | -0,2477 | 0,0001 | -0,3889 | <0,0001 |
| Haemoglobin, g/L | - | - | - | - |
| Albumin, g/L | -0,1660 | 0,0105 | -0,3628 | <0,0001 |
| Cholesterol, mmol/L | - | - | - | - |
| HDL, mmol/L | - | - | - | - |
| Triglycerides, mmol/L | - | - | 0,1854 | 0,0055 |
| Calcium, mmol/L | - | - | - | - |
| Phosphate, mmol/L | - | - | - | - |
| iPTH, ng/L | - | - | - | - |
| Creatinine, μmol/L | - | - | - | - |
| hsCRP, mg/L | 0,3067 | <0,0001 | 0,3323 | <0,0001 |
| CCB | - | - | - | - |
| Betablock | 0,2648 | <0,0001 | 0,2598 | 0,0002 |
| ACEi/ARB | - | - | - | - |
| Statin | 0,2425 | 0,0003 | 0,1820 | 0,0087 |
| sevelamer | - | - | - | - |
| Warfarin | - | - | - | - |
| Dp-ucMGP | 0,2685 | <0,0001 | 0,2682 | <0,0001 |

Abbreviations: CAC, coronary artery calcium; AVC, aortic valve calcium; CVD, cardiovascular disease; BP, blood pressure; SGA, subjective global assessment; BMI, body mass index; HGS %, hand grip strength, converted to % of sex-matched healthy controls; HDL, high-density lipoprotein; iPTH, intact parathyroid hormone; hsCRP, high sensitivity C-reactive protein; AU, Agatston units; CCB, calcium channel blockers; ACEi/ARB, angiotensin-converting enzyme inhibitor/ angiotensin II receptor blocker; dp-ucMGP, dephosphorylated-uncarboxylated matrix-Gla protein

**^*^** CAC and AVC were calculated by Agatston scoring
