# Supplementary material for: Functional vitamin K insufficiency, vascular calcification and mortality in advanced chronic kidney disease: A cohort study
Source: PLoS One. 2021 Feb 24;16(2):e0247623. doi: 10.1371/journal.pone.0247623 (PMC7904143; doi:10.1371/journal.pone.0247623)
Supplement: S3 Table — (DOCX) [file pone.0247623.s007.docx]

**S3 Table. Multivariate linear regression of factors associated with per 1-SD increase of dp-ucMGP in subgroup patients (backward stepwise selection)**

|  | **1-SD increase of dp-ucMGP**  **(CAC subgroup, total CAC^*^)** | | |  | **1-SD increase of dp-ucMGP**  **(CAC subgroup, CAC volume^#^)** | | | **1-SD increase of dp-ucMGP**  **(AVC subgroup)** | | |
| --- | --- | --- | --- | --- | --- | --- | --- | --- | --- | --- |
|  | | Coefficients | p-value | adjusted r^2^ | Coefficients | p-value | adjusted r^2^ | Coefficients | p-value | adjusted r^2^ |
| Age, per 1-SD increase | | 0.27 | <0.0001 |  | 0.27 | <0.0001 |  | 0.27 | <0.0001 |  |
| BMI, per 1-SD increase | | 0.15 | 0.01 |  | 0.15 | 0.01 |  | 0.15 | 0.01 |  |
| CCB, yes/no | | -0.23 | 0.05 |  | -0.23 | 0.05 |  | -0.23 | 0.05 |  |
| Vitamin K antagonist, yes/no | | 2.31 | <0.0001 |  | 2.32 | <0.0001 |  | 2.31 | <0.0001 |  |
| *Overall model* | |  |  | 0.34 |  |  | 0.34 |  |  | 0.34 |
| *N* | |  |  | 237 |  |  | 223 |  |  | 223 |

Abbreviations: SD, standard deviation; dp-ucMGP, dephosphorylated-uncarboxylated matrix-Gla protein; BMI, body mass index; CCB, calcium channel blockers;

* total CAC>0 as a variable in the model; # CAC volume was treated as a separate ordinal variable in the model (CAC volume 0 as reference, low and high median CAC volume).
